# Exome sequencing reveals a novel partial deletion in the progranulin gene causing primary progressive aphasia

**DOI:** 10.1136/jnnp-2013-306116

**Published:** 2013-07-31

**Authors:** Jonathan D Rohrer, Jonathan Beck, Vincent Plagnol, Elizabeth Gordon, Tammaryn Lashley, Tamas Revesz, John C Janssen, Nick C Fox, Jason D Warren, Martin N Rossor, Simon Mead, Jonathan M Schott

**Affiliations:** 1 Dementia Research Centre, Department of Neurodegenerative Disease, UCL Institute of Neurology, London, UK; 2 MRC Prion Unit, Department of Neurodegenerative Disease, UCL Institute of Neurology, London, UK; 3 Department of Statistics, Institute of Genetics, University College London, UK; 4 Queen Square Brain Bank, UCL Institute of Neurology, London, UK; 5 Department of Neurology, Chelsea and Westminster Hospital, London, UK

**Keywords:** Dementia

**Figure 1:**
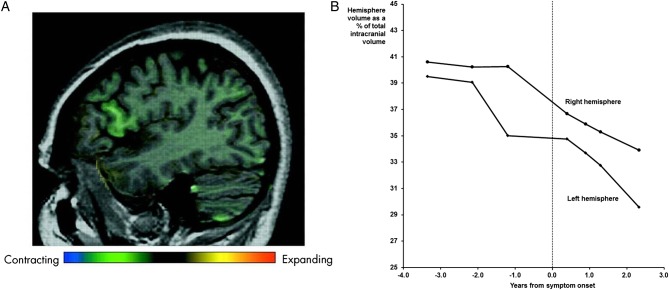
MRI changes in the proband: (A) sagittal MRI showing focal anterolateral left frontal lobe atrophy, particularly centred around the pars opercularis, using voxel compression mapping between the first and second scans (3.4 and 2.1 years prior to symptom onset) (reprinted from Janssen *et al*,^1^; (B) changes in left and right hemispheric volume over time.

In 2005, we reported a case of familial primary progressive aphasia (PPA) in this journal.^1^ The individual in question had a family history of frontotemporal dementia (FTD), her brother having behavioural variant FTD shown to be due to tau-negative, ubiquitin-positive (FTLD-U) pathology at postmortem. She was followed as part of a research programme from the age of 51 years, first developing symptoms of progressive speech disturbance at the age of 55 years. We were able to demonstrate the emergence of neuropsychometric deficits and brain atrophy prior to symptom onset. Through the use of voxel compression mapping, we showed the emergence of very focal, presymptomatic regional atrophy initially almost entirely confined to the pars opercularis (figure 1A).^1^ Over time, the atrophy spread through the frontal and temporal lobes to affect the parietal lobe and then the right frontal lobe. Subsequent analysis has shown increase in left and right hemispheric lobar atrophy prior to symptom onset, although the left hemisphere volume loss preceded and remained more prominent than the right throughout the disease course (figure 1B).

At the time of publication, mutations in the microtubule-associated protein tau (*MAPT*) were the only known genetic cause of disorders within the FTD spectrum, and screening for *MAPT* mutations was negative. Since then, the progranulin (*GRN*) and *C9ORF72* genes have been shown to be major causes of familial FTD.^2^ Her brother's pathology was subsequently reanalysed and reclassified as FTLD-transactive response DNA-binding protein (TDP) type A pathology,^3^ consistent with either a *GRN* or *C9ORF72* mutation. However, conventional analyses failed to disclose mutations in either of these genes in this family.^4–6^

**Figure 2:**
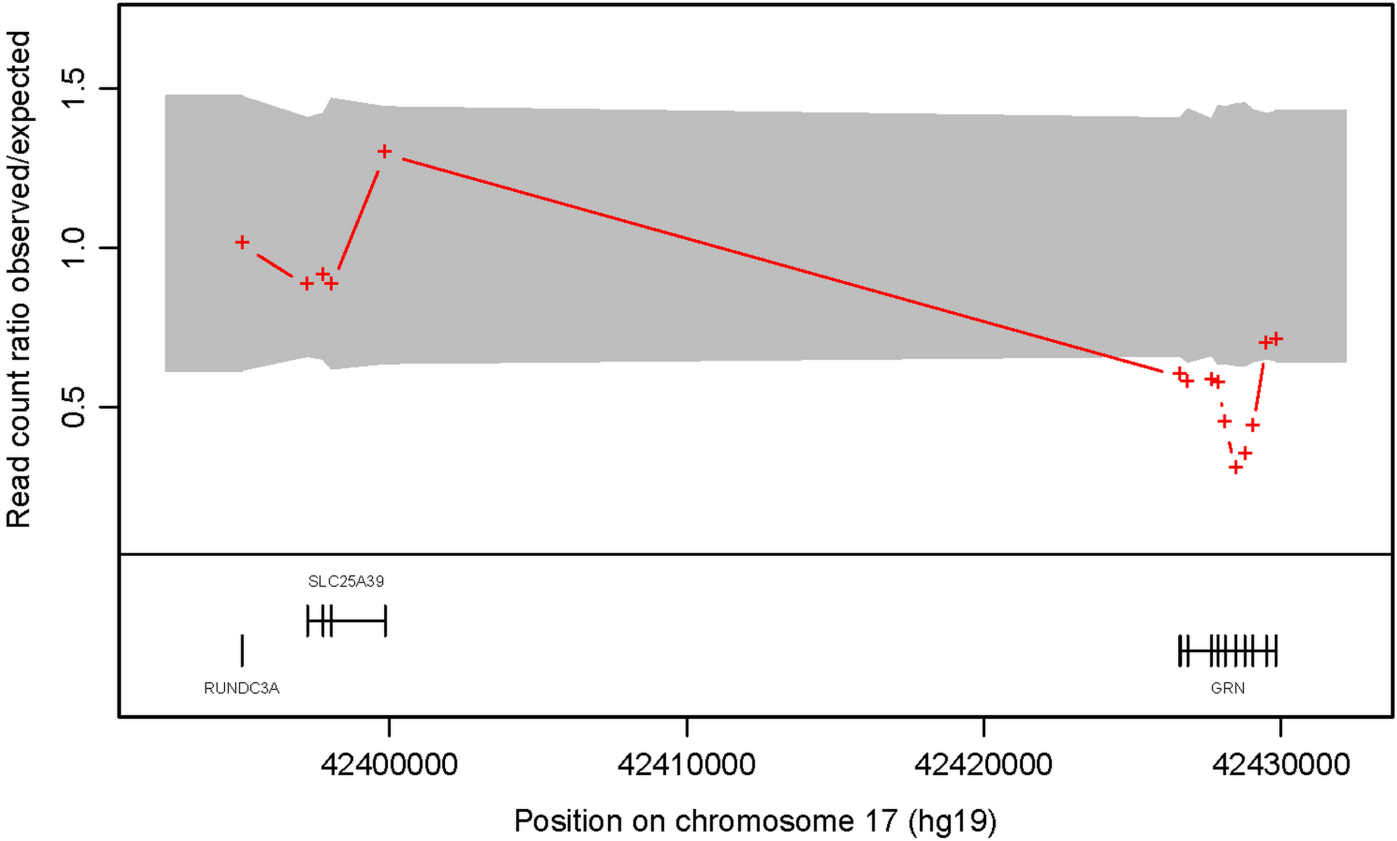
The read count ratio of observed to expected is shown plotted against position in basepairs along chromosome 17. A reduction in read count ratio to 0.5 and below at the GRN locus can be seen, and indicate a heterozygous gene deletion.

In order to investigate this further, DNA from her brother underwent exome sequencing, performed on genomic DNA using Agilent SureSelect Human All Exon v2 target enrichment kit. Sequencing was performed on an Illumina HiSeq2000 and achieved an average 30-fold depth-of-coverage of target sequence.^7^ ExomeDepth^8^ compares the read depth data between a test sample and an aggregate reference set that combines multiple exomes matched to the test sample for technical variability (Software freely available at: http://cran.r-project.org/web/packages/ExomeDepth/index.html). Analysis demonstrated a *GRN* gene deletion. The red crosses (figure 2) show the ratio of observed/expected number of reads for the test sample. The grey shaded region shows the estimated 99% CI for this observed ratio in the absence of copy number variation (CNV) call. The presence of contiguous exons with read count ratio located outside of the CI is indicative of a heterozygous deletion in the *GRN* gene. *GRN* exons 0, 2, 5, 9 and 11 were subsequently probed for copy number variation using multiplex ligation-dependent probe amplification (MLPA) analysis with the Medical Research Council (MRC) Holland kit P275, which is routinely used for assessing *GRN* deletions.^9^ ^10^ This confirmed the presence of a novel heterozygous deletion of exons 2–11. Test results show the 5′ untranslated region of the gene was present, but could not determine if exon 12 of *GRN* was also deleted. The same deletion was detected in the proband using MLPA analysis.

While progranulin mutations were not known to cause frontotemporal lobar degeneration at the time of our original report, the clinical features that emerged during the course of her illness would now be recognised as being fairly characteristic. Progranulin mutations are usually associated with behavioural variant FTD or PPA,^2^ ^3^ with combinations of these presentations recognised within the same family. Neuropsychologically, patients often have executive dysfunction and early parietal lobe deficits, with PPA patients having a non-fluent aphasia with a prominent anomia. Imaging studies in patients with established disease typically show prominent asymmetrical atrophy affecting frontal, temporal and parietal lobes consistent with neuropsychological findings.^11^ Finally, the pathology, type A TDP-43, would be consistent with that seen in progranulin mutations (and also C9ORF72 expansions).^2^ ^3^

This case serves to illustrate a number of important points. First, progranulin mutations should always be considered as a cause of PPA where there is a positive family history either of PPA or behavioural variant FTD. Second, prominent asymmetric lobar atrophy on MRI is an important clue to the presence of a progranulin rather than a MAPT or C9ORF72 mutation in the context of an autosomal-dominant family history. Third, as with other neurodegenerative diseases, this case shows that focal brain atrophy precedes symptom onset in genetically determined forms of FTD; rates of atrophy may therefore be useful outcome measures for presymptomatic therapeutic trials in these disorders. Finally, the fact that conventional progranulin testing was negative in this case demonstrates the power of exome sequencing as a tool to discover large-scale mutations, such as the partial deletion seen here, that may not be found with usual screening methods for small-scale changes.
